# Cerebrospinal Fluid of Patients With Alzheimer’s Disease Contains Increased Percentages of Synaptophysin-Bearing Microvesicles

**DOI:** 10.3389/fnagi.2021.682115

**Published:** 2021-07-06

**Authors:** Janine Utz, Judith Berner, Luis Enrique Muñoz, Timo Jan Oberstein, Johannes Kornhuber, Martin Herrmann, Juan Manuel Maler, Philipp Spitzer

**Affiliations:** ^1^Department of Psychiatry and Psychotherapy, Friedrich-Alexander-University of Erlangen-Nuremberg (FAU), Erlangen, Germany; ^2^Department of Internal Medicine, Friedrich-Alexander-University of Erlangen-Nuremberg (FAU), Erlangen, Germany; ^3^Department of Rheumatology and Immunology, Friedrich-Alexander-University of Erlangen-Nuremberg (FAU), Erlangen, Germany

**Keywords:** Alzheimer’s disease, synaptophysin, microvesicle, biomarker, synaptic loss, extracellular vesicles, cerebrospinal fluid, tau

## Abstract

**Introduction:**

In Alzheimer’s disease, the severity of symptoms is linked to a loss of synaptic density and the spread of pathologically hyperphosphorylated tau. The established cerebrospinal fluid markers Aβ, tau and phospho-tau reflect the histopathological hallmarks of Alzheimer’s disease but do not indicate disease progression. Such markers are of special interest, especially for trials of disease modifying drugs. Microvesicles are produced by stressed cells and reflect part of the metabolism of their cells of origin. Therefore, we investigated microvesicles of neuronal origin in cerebrospinal fluid.

**Materials and Methods:**

We used flow cytometry to analyze microvesicles carrying tau, phospho-tau-Thr181, phospho-tau-Ser202Thr205, synaptophysin, and SNAP-25 in the cerebrospinal fluid of 19 patients with Alzheimer’s disease and 15 non-inflammatory neurological disease controls.

**Results:**

The percentages of synaptophysin-bearing microvesicles were significantly higher in the cerebrospinal fluid of patients with Alzheimer’s disease than in the CSF of non-inflammatory neurological disease controls. Tau, phospho-tau-Thr181, phospho-tau-Ser202Thr205, and SNAP-25 did not differ between the groups. The percentages of synaptophysin-bearing vesicles distinguished patients with Alzheimer’s disease from the controls (AUC = 0.81).

**Conclusion:**

The loss of synapses in Alzheimer’s disease may be reflected by synaptophysin-bearing microvesicles in the cerebrospinal fluid. Future studies are needed to investigate the possibility of using these MVs as a marker to determine the activity of Alzheimer’s disease.

## Introduction

While the pathological hallmarks of Alzheimer’s disease (AD)–amyloid plaques, neurofibrillary tangles, and cortical atrophy–have been known for decades, the underlying pathological mechanism is still elusive (Strooper and Karran, 2016; Guo et al., 2020). The severity of clinical symptoms is correlated with the loss of synapses and the spread of pathologically hyperphosphorylated tau in the brain (DeKosky and Scheff, 1990; Terry et al., 1991; Clare et al., 2010). This change is not represented by the established CSF biomarkers Aβ, tau, and phospho-tau (DeKosky and Scheff, 1990; Terry et al., 1991; Arriagada et al., 1992; Clare et al., 2010; Drago et al., 2011; Molinuevo et al., 2018; Rabbito et al., 2020).

Microvesicles (MVs) are currently under intense investigation as biomarkers in neurodegenerative diseases, such as Parkinson’s disease, multiple sclerosis, and AD (Ciregia et al., 2017; Watson et al., 2019; Aharon et al., 2020; Badhwar and Haqqani, 2020). MVs are produced by virtually all mammalian cells (Freyssinet, 2003), and along with exosomes and apoptotic bodies, they belong to the extracellular vesicle (EV) family. Apoptotic bodies originate from cells undergoing apoptosis and reportedly measure more than 1,000 nm (Dozio and Sanchez, 2017). Most exosomes are smaller than 100 nm, are excreted from multivesicular endosomes by their cells of origin, and they carry the tetraspanin CD9, among other proteins, on their surfaces (Andreu and Yáñez-Mó, 2014; Dozio and Sanchez, 2017). MVs are of intermediate size (100–1,000 nm) and originate from budding of the membrane (Raposo and Stoorvogel, 2013). While most studies refer to a specific subtype of EV, the nomenclature used in the literature is not always consistent (Teng and Fussenegger, 2020). Further experimental work is needed, especially in the classification of the respective subpopulations of exosomes, MVs and apoptotic bodies (Yáñez-Mó et al., 2015).

Microvesicles are detectable in varying amounts in nearly all bodily fluids, e.g., blood, cerebrospinal fluid (CSF), and urine (Raposo and Stoorvogel, 2013; Zaborowski et al., 2015). While MVs were long viewed as a mere method of waste disposal, evidence has emerged that they have additional (patho)physiological functions as intercellular messengers (van Niel et al., 2018). The formation of MVs, while not yet fully understood, involves several mechanisms. MVs vary in their composition, but they often carry contents of membrane lipid rafts. The distribution of lipids in the cell membrane seems to determine where and when membrane budding occurs (Janas et al., 2016). Several molecular mechanisms of MV biogenesis have been described. Some are common to the formation of exosomes, e.g., the ESCRT machinery (Teng and Fussenegger, 2020), but several other proteins are also involved, such as translocases and scramblases, which interact with phospholipids, as well as enzymes that interact with the cytoskeleton (You and Ikezu, 2019). Since they are produced via plasma membrane budding, MVs may carry select surface antigens from their cells of origin and may contain mRNA, proteins and other components of cellular metabolism (Abels and Breakefield, 2016). The contents of MV subpopulations are understood to be highly regulated and dependent on the cell of origin. Complex sorting occurs and leads to the enrichment of MVs with different RNA species and cytosolic proteins (You and Ikezu, 2019). The properties and functions of these MVs are slowly being revealed. Their functions include neurodevelopment (Gomes et al., 2020), cell-to-cell communication, and even synaptic transmission (Krämer-Albers and Hill, 2016). Along with exosomes, MVs are discussed as vehicles for the intercellular spread of pathologically hyperphosphorylated tau (Sharma et al., 2013; Coleman and Hill, 2015; Quek and Hill, 2017; Ruan et al., 2021). When discussing AD, a disease with substantial neuroinflammation, it is important to keep in mind that MVs are increasingly produced by stressed cells (Yamamoto et al., 2016) and that cellular stress can change their content (Yáñez-Mó et al., 2015).

Regarding the investigated targets, synaptophysin may serve as a marker of the degeneration of synapses (Gudi et al., 2017). It is abundant at the synaptic site of neurons and is used to quantify synapses (Clare et al., 2010). Synaptosome-associated protein of 25,000 daltons (SNAP-25) is another synaptic protein (Clare et al., 2010) that is found on exosomes in the CSF (Agliardi et al., 2019) and has been described as a marker of synaptic degeneration in AD (Brinkmalm et al., 2014).

The intraneuronal protein tau is commonly regarded as a surrogate for neurodegeneration. It is pathologically hyperphosphorylated in AD, where it tends to agglutinate and form pathognomonic neurofibrillary tangles (Goedert et al., 2017). It has been suggested that pathological tau variants interfere with the function of synapses and with the physiological release of synaptic vesicles (Naseri et al., 2019).

In this study, we investigated the possibility of using MV-borne proteins as markers of AD. We sought to determine whether hyperphosphorylated tau can be found in MVs from CSF. Furthermore, we quantified the amount and composition of MVs carrying tau, phospho-tau-Thr181, and phospho-tau-Ser202Thr205 as well as the neuronal proteins SNAP-25 and synaptophysin.

## Materials and Methods

### Patients

Patients with cognitive complaints were recruited at the memory clinic of the Department of Psychiatry, University Hospital Erlangen. The study protocol was approved by the Ethical Committee of the University Hospital (Nr. 3987), and patients or their legal guardians provided informed written consent.

The patients participated in a psychiatric consultation, neurological and medical examination, as well as neuropsychological testing with the CERADplus test battery (Morris et al., 1989) and received an MRI scan. In our study, CSF and blood samples were also obtained for routine neurochemical dementia diagnostics.

Alzheimer’s disease was diagnosed according to the NINCDS-ADRDA criteria using the Aβ42/Aβ40 ratio as well as the tau and phospho-tau levels in the CSF (Albert et al., 2011; McKhann et al., 2011). The CSF biomarkers were interpreted in accordance with the criteria of the Erlangen Score (ES) algorithm (Lewczuk et al., 2015; Somers et al., 2019). Patients with dementia or mild cognitive impairment (MCI) with high evidence of AD pathophysiological processes (AD group; Erlangen Score ≥ 3; *n* = 19) and patients without evidence of AD pathophysiological processes (non-inflammatory neurological disease control group CON; Erlangen Score ≤ 1; *n* = 15) were included. This also follows the criteria suggested by Jack et al: Abeta (A) and phospho-tau (T) positivity was determined according to their levels in the CSF, and neurodegeneration (N) was determined either by atrophy as seen on the MRI scan or by elevated CSF tau levels. Patients in the AD group were A+/T+/N+, whereas those in the control group were A−/T−/N− (Jack et al., 2016). Patients with intermediate signs of AD pathophysiology were excluded.

### Sample Processing and Storage

Cerebrospinal fluid in polypropylene tubes was centrifuged at 750 g for 5 min within 30 min of sample acquisition. The supernatant containing the MVs was aliquoted and stored at −80°C until further use.

### Antibody Staining for Flow Cytometry

Cerebrospinal fluid was thawed (3 min at 37°C) and immediately incubated with human polyvalent immunoglobulin (Beriglobin, CSL Behring, Marburg, Germany) for 20 min at room temperature (RT) to avoid non-specific antibody binding. This protocol leaves the MVs intact but also permeabilizes them for staining of intracellular epitopes (Spitzer et al., 2019). Primary antibodies were added, and the samples were incubated at 4°C for 30 min and then centrifuged at 16,000 rpm for 15 min at 4°C. All primary antibodies were diluted 1:1,000 in PBS (MERCK, Darmstadt, Germany). We used anti-tau1-AF488 (clone P1C6, Merck-Millipore, Darmstadt, Germany), anti-phospho-tauThr181-AF488 (clone C11107, SAB Biotech, College Park, MD, United States), and anti-SNAP-25-FITC (clone SP12, Thermo Fisher Scientific, Waltham, MA, United States) as labeled primary antibodies. Anti-phospho-tauSer202Thr205-Biotin (AT8, Thermo Fisher Scientific, Waltham, MA, United States) was visualized with Streptavidin-BD-Horizon-BB515 (1:1,000; BD Pharmingen, Heidelberg, Germany). Anti-synaptophysin (SY38, mouse IgG1, Thermo Fisher Scientific, Waltham, MA, United States) was stained with goat anti-mouse AF488 IgG (1:10,000, Thermo Fisher Scientific, Waltham, MA, United States) as a secondary antibody. If secondary antibodies were added, they were incubated for another 30 min at 4°C, after which the sample was centrifuged. Additionally, we used CD9-PerCP-Cy5.5 (M-L13, BD Pharmingen, Heidelberg, Germany) staining in all samples to discriminate between exosomes and MVs.

### Flow Cytometry Analysis

Unstained samples were used to determine the background fluorescence and the total number of MVs (Chandler, 2016). MVs were gated by size with a forward scatter below the size of 1 μm polystyrene beads (Sigma-Aldrich, Steinheim, Germany), excluding the background signal. In stained samples, fluorescent events were distinguished from negative events and were gated accordingly (Trummer et al., 2008; Supplementary Figure 1). To control for varying sample volumes and irregularities in the flow, a standard quantity of 3 μm APC-labeled beads (Sysmex Partec GmbH, Görlitz, Germany) was added to each sample. If the measured number of beads differed more than 30% from the expected value, the sample was excluded from the analysis.

Samples were measured undiluted using a Gallios flow cytometer (Beckman and Coulter, Brea, CA, United States) and analyzed with Kaluza 2.1 software (Beckman and Coulter, Brea, CA, United States). If the number of total events in a sample were extremely divergent from the other samples, e.g., if the CSF was contaminated with blood or other particles, those samples were excluded from further analysis.

### Statistical Analysis

Statistical analysis was performed using Prism 5.0 (GraphPad Software, La Jolla, CA, United States). The number of positive MVs was standardized to the number of all MVs in the same sample to account for the interindividual variability in the number of extracellular vesicles.

Since most data were not normally distributed, we applied the non-parametric two-sided Mann-Whitney *U*-test to compare the groups. Correlations were calculated using the Spearman test. To evaluate the discriminatory ability of the markers, receiver operating characteristic (ROC) curves were used. The results are presented as medians with interquartile ranges and were considered significant at a *p*-value <0.05.

## Results

Only significant values are displayed in the results section, unless otherwise stated.

### Patients

Patients in this study were referred to the memory clinic of the Department of Psychiatry of the University Hospital Erlangen. Only patients with conclusive clinical and biomarker workups were included. Nineteen patients were included in the AD group and were positive for the Aβ42/Aβ40 ratio, tau, and phospho-tau. All patients experienced continuous cognitive decline, showed an amnestic phenotype in neuropsychological testing, and exhibited no clinical or biomarker signs of other neurodegenerative diseases. Regarding symptom severity, three patients were in the stage of MCI, 11 were in the stage of mild clinical dementia, and five had severe dementia.

The disease controls consisted of 15 patients with neurological disease without biomarker evidence of AD (one patient with normal pressure hydrocephalus, three patients with vascular dementia, four patients with frontotemporal dementia, four patients with depression, one patient with schizophrenia, one patient with epilepsy, one patient with subjective cognitive complaints). According to the criteria suggested by Teunissen et al., patients in this group were considered to be non-inflammatory neurological disease controls (Teunissen et al., 2014).

Since samples were collected prospectively, the patients were not age-matched. The median age was higher in the AD group (73.3 years) than in the control group (65.1 years, Table 1; *p* = 0.0061). Patients in the control group performed better on the Mini Mental State Examination (MMSE; *p* = 0.0129). No differences were observed between the groups in terms of Beck’s Depression Inventory (BDI; *p* = 0.1742) or the clock drawing test (CDT; *p* = 0.2820). The CSF showed no differences in the albumin quotient (Table 1; *p* = 0.3185) or erythrocyte count (Table 1; *p* = 0.2345).

**TABLE 1:** Patient characteristics.

	Disease controls (CON)	Alzheimer’s disease (AD)
*N* (female)	15 (7)	19 (9)
Age	65.14 [56.00–70.78]**	75.31 [68.96–79.02]**
MMSE	27.00 [22.75–29.25]*	23.00 [17.00–26.00]*
CDT	2.0 [1.0–4.0]	3.0 [2.0–4.0]
BDI	14.00 [4.75–19.00]	6.75 [2.00–13.50]
CSF erythrocytes (/μl)	0.00 [0.00–1.00]	1.00 [0.00–19.00]
Albumin quotient	4.96 [4.49–5.97]	6.30 [4.47–7.77]

Patient characteristics are shown as the median [interquartile range]. Differences between the groups were calculated using the non-parametric Mann-Whitney U test.*p < 0.05; **p < 0.01.AD, Alzheimer’s disease; CON, control; MMSE, Mini Mental State Examination; CDT, clock drawing test; BDI, Beck Depression Inventory; CSF, cerebrospinal fluid.

### The Percentages of Synaptophysin-Bearing MVs Were Elevated in the AD Group

**FIGURE 1:**
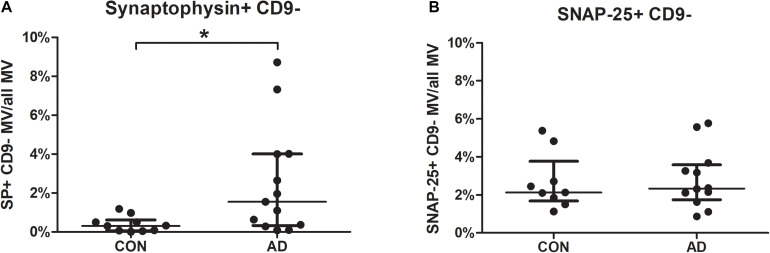
Percentages of CSF-borne synaptophysin-bearing microvesicles are increased in patients with Alzheimer’s disease (AD). MVs in the CSF of 19 AD patients and 15 controls were gated as shown in Supplementary Figure 1, and samples were excluded if they contained high amounts of cell debris. The percentages of synaptophysin- **(A)** and SNAP-25- **(B)** bearing MVs are shown for AD patients and controls. Differences between the groups were calculated using the non-parametric Mann-Whitney *U* test. *p < 0.05 (SP: *p* = 0.0143; SNAP-25: *p* = 0.6959; AD, Alzheimer’s disease; CON, disease controls; CSF, cerebrospinal fluid; MVs, microvesicles; SP, synaptophysin; SNAP-25, synaptosome-associated protein of 25,000 daltons).

Focusing on synaptic markers, the percentages of synaptophysin-bearing MVs were higher in the CSF of the AD group than in the CSF from the control group (*p* = 0.0143). Samples of 19 AD patients and 15 non-inflammatory neurological disease controls were measured. After samples contaminated with cell debris were excluded, 13 AD and 10 control samples were analyzed for synaptophysin. Agglutinated exosomes and MVs could be distinguished by staining for CD9. The mean fluorescence intensity of the MVs did not differ (AD median = 3.91 vs. CON median = 2.72; *p* = 0.204). This indicates the presence of a higher number of synaptophysin-bearing MVs but not a higher concentration of synaptophysin per MV (Figure 1A).

**FIGURE 2:**
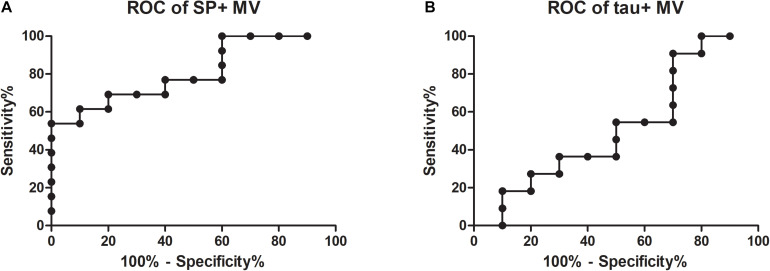
Receiver operating characteristic (ROC) curves for the percentages of synaptophysin- and tau-bearing microvesicles in CSF. ROC values were calculated for the discrimination between Alzheimer’s disease and disease controls according to the numbers of synaptophysin-bearing MVs (**A**; AUC = 0.81) and tau-bearing MVs (**B**; AUC = 0.52; AUC, area under the curve; SP, synaptophysin; MVs, microvesicles).

Receiver operating characteristic values were calculated to test whether synaptophysin-bearing MVs are a suitable biomarker for AD. Patients with high (*n* = 13) and no (*n* = 10) evidence of AD pathology could be discriminated (Figure 2A; AUC = 0.81; for comparison, the ROC of tau + MV is shown in Figure 2B with an AUC = 0.52). According to a Spearman test, for both groups, the percentages of synaptophysin-bearing MVs correlated neither with the performance on the MMSE nor with age.

### SNAP- 25-, Tau- and Phospho-Tau-Bearing MVs Remain Unchanged

**FIGURE 3:**
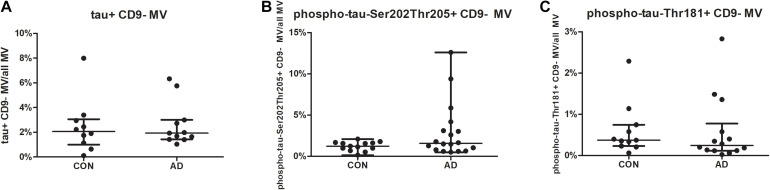
The percentages of tau-/phospho-tau-bearing microvesicles are similar in AD and disease controls (CON). MVs in the CSF of 19 AD patients and 15 controls were gated as shown in Supplementary Figure 1, and samples were excluded if they contained high amounts of cell debris. The percentages of tau- **(A)**, phospho-tau-Ser202Thr205- **(B)**, and phospho-tau-Thr181- **(C)** bearing MVs are shown for AD patients and controls. Differences between the groups were calculated using the non-parametric Mann-Whitney *U* test. *p < 0.05 (tau: *p* = 0.9159, phospho-tau-Ser202Thr205: *p* = 0.2221 and phospho-tau-Thr181: *p* = 0.2857; AD, Alzheimer’s disease; CON, control; CSF, cerebrospinal fluid; MVs, microvesicles).

For SNAP-25, no differences were found between the groups for neither the mean fluorescence intensity nor the percentages of SNAP-25-bearing MVs or exosomes (Figure 1B). For the tested phospho-tau variants (phospho-tau-Ser202-Thr205; phospho-tau-Thr181) and tau, no differences were found in the mean fluorescence intensities or the percentages of (phospho-) tau-positive CD9-negative MVs between the groups (Figure 3).

## Discussion

In this study, we found that the percentages of synaptophysin-bearing MVs were increased in the CSF of AD patients compared with the CSF of non-AD patients. Interestingly, the mean fluorescence intensity was similar in both groups. This shows that more synaptophysin-bearing MVs are produced in AD but that the amount of synaptophysin per individual MV remains unchanged. Synaptophysin is expressed by neurons at the synaptic site and is a marker of synapses (Clare et al., 2010). This implies a higher production of synaptic MVs. Since MVs are increasingly produced by stressed cells and cellular stress is a key component of AD (Strooper and Karran, 2016), AD pathology at least partially occurs at synapses. As mentioned above, the biogenesis of MVs is not satisfactorily understood (You and Ikezu, 2019). The design of sound cell culture models is challenging, especially when investigating the interaction of different cell types in the brain, but it has been achieved (Pascua-Maestro et al., 2018; Gharbi et al., 2020; Kriaučiūnaitė et al., 2021). To gain insights into synaptophysin-bearing MVs in AD that are applicable to *in vivo* conditions, cell culture models of human AD neurons, which do not currently exist, are needed. Nevertheless, this fundamental research is vital to understanding clinical findings such as ours.

Synapses are of special interest in AD since the severity of clinical symptoms correlates with the total loss of synapses in the brains of patients (DeKosky and Scheff, 1990; Clare et al., 2010). Neuronal loss is usually evaluated according to postmortem protein analysis of brain tissue, which indicates the stage of AD (Clare et al., 2010; Hong et al., 2016; Yamazaki et al., 2019). *In vivo* markers showing the loss of synapses are valuable tools and serve as surrogates for cognitive decline (Blennow and Zetterberg, 2015). Established CSF markers can identify AD and predict conversion from MCI to AD (Drago et al., 2011). Longitudinal studies show that currently established markers react early but are not sensitive to the cognitive decline observed at later points (Fagan et al., 2014). However, it is important to have a marker of AD activity especially when investigating the interaction of different cell types in the brain (Blennow and Zetterberg, 2015; Hill, 2019; Colom-Cadena et al., 2020). Consistently, Bhargava et al. (2020) proposed using extracellular vesicles as a marker of neuronal loss in multiple sclerosis.

All our non-inflammatory neurological disease controls were patients with cognitive complaints and were not healthy individuals. This means that there was a clinical need for differential diagnosis between our sample groups, which is often difficult. It is even difficult to discriminate between AD and other causes of dementia, such as progressive non-fluent aphasia or Lewy body dementia, using the established diagnostic markers (Paterson et al., 2018). For the discriminatory ability of synaptophysin-bearing MVs, we calculated an AUC value of 0.81 between the AD group and the non-inflammatory neurological disease control group. The increased percentages of synaptophysin-bearing MVs found in this study were not linked to MMSE performance and thus a state of cognitive decline. The way in which synaptophysin-bearing MVs are generated implies that they are also markers of disease activity and not merely a marker of diagnostic traits (Marostica et al., 2020). To verify this, follow-up samples with respective MMSE scores must be analyzed in future studies. For a physiological understanding, a comparison to healthy, age-matched controls would be important.

Microvesicles are of interest as diagnostic markers. Changes in MVs in CSF have been observed in several neurological disorders, such as multiple sclerosis and stroke (Colombo et al., 2012; Ciregia et al., 2017). Recent studies have shown that MVs can pass through the blood brain barrier and can be measured in blood samples (García-Romero et al., 2017; Mustapic et al., 2017). Further basic research is needed to understand under which circumstances this occurs. Since the established CSF biomarkers for AD (Aβ, tau, and phospho-tau) require lumbar puncture, it is desirable to find biomarkers that are more conveniently accessible (Humpel, 2011). Contamination with peripheral neuronal MVs and the high load of membrane-coated particles in the blood complicate these investigations (Chandler, 2016; Lee et al., 2019). If these methodological challenges can be overcome, this will open a plethora of new possibilities. Interesting findings regarding exosomes of central nervous system origin, for which analysis of blood-derived samples is better understood (Li et al., 2019), have already been reported. Fiandaca et al. (2015) detected exosomes of neuronal origin carrying phospho-tau-Thr181 in the blood of patients with AD. Goetzl et al. (2016) reported that blood-derived synaptophysin-bearing exosomes are already altered years before AD patients display overt clinical symptoms. When the detection of blood-derived MVs is established, study collectives can be broadened because of easier sample acquisition. Since MVs can be analyzed using flow cytometry, whereas exosomes cannot (Welsh et al., 2020), the advantages of this method will provide further insight into the pathology of AD, as reflected by EVs. The possibility of quantifying several antigens in the same sample and relating them to the size and granularity of the particles allows for more nuanced investigations (Marostica et al., 2020). It may also be possible to identify subpopulations of MVs and further explore their function in AD (Andjus et al., 2020; Ratajczak and Ratajczak, 2020; Veziroglu and Mias, 2020).

In this study, we did not find differences in the content of tau or phospho-tau in MVs. Thus, tau-bearing MVs are not suitable biomarkers for the identification of AD patients. Interestingly, it has been found that the tau oligomer concentration is increased in CSF exosomes in AD (Ruan et al., 2021). This led to the suggestion that tau is secreted via exosomes and that this is a mechanism for the pathological spread of tau between neurons in AD (Saman et al., 2012; Wang et al., 2017). These findings indicate that MVs, such as those in our study, and exosomes can behave very differently, which highlights the importance of discriminating between the different types of EV cargo, e.g., tetraspanins such as CD9.

Limitations of this study include the small patient cohort. Repeating the experiments with a larger sample size is necessary to further validate these results. The age difference between patients in the AD and CON cohorts, caused by the prospective collection of samples, must be addressed in future studies. A strength of this study was that while the sample size was small, the patients were well characterized by biomarkers and clinical workups. We controlled for several neuropsychological components, including MMSE, CDT, and BDI, as well as biological variables, such as CSF erythrocyte count and CSF albumin ratio. The discrimination between MVs and agglutinated exosomes using CD9 staining allowed for a more accurate differentiation between populations of EVs.

## Conclusion

We conclude that the percentages of synaptophysin-bearing MVs in CSF potentially reflect the loss of synapses in AD. While these are limited findings, these MVs could become a valuable *in vivo* marker of disease activity in trials testing disease modifying drugs, if validated in future studies and explored by further groundwork.

## Data Availability Statement

The datasets generated and/or analyzed in the current study are available from the corresponding author on reasonable request.

## Ethics Statement

The studies involving human participants were reviewed and approved by Ethical Committee of the University Hospital Erlangen. The patients/participants provided their written informed consent to participate in this study.

## Author Contributions

JU, JB, MH, JK, PS, and JM designed the study. JU, JB, and LM performed the experiments. JU, JB, LM, TO, MH, JK, PS, and JM analyzed and interpreted the data. JU and PS performed the statistical analysis. JU, TO, PS, and JM drafted the article. All authors critically reviewed the manuscript and provided constructive comments to improve the quality of the manuscript. All authors have read and approved the final manuscript.

## Conflict of Interest

The authors declare that the research was conducted in the absence of any commercial or financial relationships that could be construed as a potential conflict of interest.
