# Supplementary figures and images for: Cerebrospinal Fluid of Patients With Alzheimer’s Disease Contains Increased Percentages of Synaptophysin-Bearing Microvesicles
Source: Front Aging Neurosci. 2021 Jul 6;13:682115. doi: 10.3389/fnagi.2021.682115 (PMC8290128; doi:10.3389/fnagi.2021.682115)

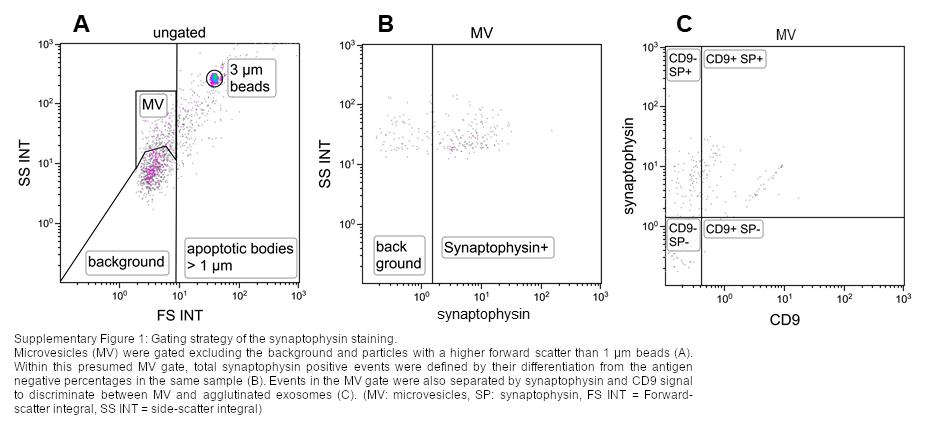

Supplement: Supplementary file 1 [file Image_1.TIF]
